# Genetic Polymorphisms of rs3077 and rs9277535 in HLA-DP associated with Systemic lupus erythematosus in a Chinese population

**DOI:** 10.1038/srep39757

**Published:** 2017-01-17

**Authors:** Junlong Zhang, Wenli Zhan, Bin Yang, Anning Tian, Lin Chen, Yun Liao, Yongkang Wu, Bei Cai, Lanlan Wang

**Affiliations:** 1 Department of Laboratory Medicine, West China Hospital, Sichuan University, Chengdu, Sichuan Province, China

## Abstract

Although the SLE risk gene loci of *HLA-DR* and *HLA-DQ* within the major histocompatibility complex (MHC) region has been gradually revealed by recent Genome-Wide Association studies (GWAS), the association of *HLA-DP* polymorphisms with SLE was minimally reported. Considering that the variants in rs3077 and rs9277535 in the *HLA-DP* region could influence the immune response by affecting antigen presentation of HLA class II molecules to CD4^+^ T cells, the present study aimed to explore the role of *HLA-DP* polymorphisms in SLE. In total, samples from 335 SLE patients and 635 healthy controls were collected and genotyped by a polymerase chain reaction-high resolution melting (PCR-HRM) assay. A significant positive correlation was observed between the SNP rs3077, rs9277535 of *HLA-DP* and SLE susceptibility (rs3077, OR = 0.74, 95%CI = 0.60–0.91, *P* = 0.004; rs9277535, OR = 0.72, 95%CI = 0.59–0.88, *P* = 0.001). Rs3077 polymorphism was corelated to IL-17, INF-γ and cutaneous vasculitis (*P* = 0.037, *P* = 0.020 and *P* = 0.006, respectively). Additionally, rs3077 AA genotype carriers showed lower concentration of inflammatory cytokines and lower cutaneous vasculitis incidence than did the other two genotype. No significant association was observed between rs9277535 and cytokines or any clinical features. In conclusion, *HLA-DP* polymorphisms (rs3077 and rs9277535) were associated with SLE susceptibility and the levels of some inflammatory cytokines in SLE patients.

Systemic lupus erythematosus (SLE) is a multisystem autoimmune disorder characterized by a lack of tolerance to self-antigens and the hyper production of multiple pathogenic autoantibodies and immune complexes, which result in systemic inflammation and damage to multiple organ systems^1^. With significant morbidity, disability, and mortality rates, the quality of life and life expectancy in SLE patients are notably influenced^1^,^2^,^3^. The estimated incidence rates of SLE distribute differently in Asia, America and Europe, varying from 0.001% to 0.097%^4^,^5^. In China, the prevalence of SLE is 0.03% and it’s one of the most common chronic diseases^6^. However, the etiology of SLE has not yet been clarified. Among the well-known predisposing variables such as environmental, infection and hormonal factors, it has been established that genetic factors have pivotal effects on susceptibility to SLE^7^,^8^,^9^.

With the application of genome-wide association and independent replication studies, more than 40 robust genetic associations with SLE have been identified, and the gene variants in the major histocompatibility complex (MHC) region are believed to contribute the greatest genetic risk for SLE^10^,^11^,^12^,^13^. MHC region is about 4 Mb, divided into three subregions, namely class I (*HLA-A, B, C*), class II (*HLA-DR, DP, DQ*) and class III (*C2 et al.*) region. The genes which encode glycoproteins that process and present peptides for T cells recognition fall under classes I and II, while other immune genes like *C4A and C4B* located within the class III region. Although the long-range linkage disequilibrium (LD) within the MHC region makes the disease susceptibility contribution of each component gene difficult to assess, overwhelming evidence confirmed that the genetic variants in *HLA-DR* and *HLA-DQ* are predisposed to SLE^14^,^15^. Through transancestral mapping of the MHC region, allele variants in *HLA-DPB1* were also shown associated with SLE^16^. Besides, HLA-DPB1*05:01 was reported relating to the presence of autoantibodies in Japanese SLE patients^17^. However, these studies neither have found the role of allele variants on *HLA-DPA1* nor have explored the further association between the reported SNPs and the process of SLE.

Additionally, considering that a number of studies have revealed that *HLA-DP* genes were associated with susceptibility to many autoimmune diseases, including Wegener’s granulomatosis, systemic sclerosis, multiple sclerosis and others^18^,^19^,^20^, *HLA-DP* polymorphisms are likely to play an important role in SLE. In addition, recent Genome-Wide Association studies (GWAS) validated that rs3077 and rs9277535 genetic variants in the *HLA-DP* locus were significantly associated with HBV infection in Asian population^21^. Due to the fact that *HLA-DP* genes encode antigen-binding sites and exon 2 of *HLA-DPs* is highly polymorphic, the variants of rs3077 and rs9277535 may regulate the immune responses to infection by means of affecting the function of antigen presentation of HLA class II molecules in immune cells.

Meanwhile, antigen presentation is related to CD4^+^ T cells directly. And active CD4^+^ T cells, which could differentiate into disparate T cells subsets, play a critical role in development of SLE by secreting all kinds of cytokines, such as inflammatory cytokines (IL-1β, IL-6, INF-γ, TNF-α and IL-17), or the inhibitory cytokine IL-10. Serum INF-γ was primarily derived from Th1 cells, IL-6 and IL-10 were mainly from Th2 cells and IL-17 were from Th17 cells. Some studies have shown that these cytokines influenced SLE^22^.

Therefore, in the present study, we aim to explore the association of the *HLA-DP* polymorphisms rs3077 and rs9277535 with SLE susceptibility and further investigate the influence of *HLA-DP* polymorphisms rs3077 and rs9277535 on critical serum cytokine levels and clinical features in SLE in Chinese Han nationality.

## Results

### Characteristics of the included subjects

The primary demographic and clinical characteristics were illustrated in Table 1. A total of 335 SLE patients and 635 health people participated in the study. The age and sex of the participants from two groups were matched (*P* = 0.795 and 0.480, respectively). The average age of SLE patients and health controls were 35.77 and 36.28 years old, respectively. In SLE patients, the average disease duration was 36.00 (12.00–96.00) months and the average SLEDAI score was 11.76 ± 5.53. Among the SLE group, ANA, anti-dsDNA and anti-Sm antibody positive frequencies were 91.94%, 35.82% and 28.66%, respectively; while the average serum C3 concentration was 0.67 ± 0.30 g/L, and the average C4 was 0.15 ± 0.08 g/L. Additionally, the prevalences of cutaneous vasculitis, arthritis, albuminuria, rash, pleuritis, pericarditis and neurological disorder were 9.55%, 39.40%, 74.63%, 29.85%, 1.49%, 1.49% and 9.85%, respectively.

### Genotyping and LD evaluation

All participants were genotyped via the PCR-HRM methods for SNPs rs3077 and rs9277535 in the *HLA-DP* region. The veracity of the results was confirmed by direct sequencing of PCR products from randomly selected samples. The direct sequencing results were consistent with all of the corresponding genotyping results. All genotypes were distributed in concordance with the Hardy-Weinberg equilibrium (HWE), as determined at the 0.05 significance level.

**Figure 1:**
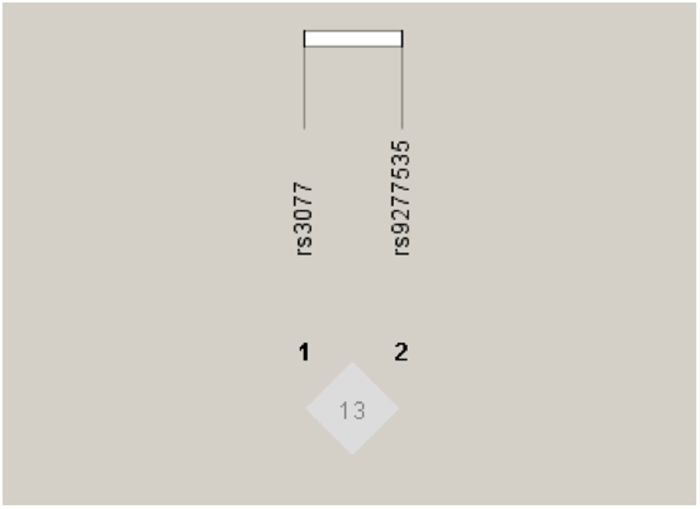
Linkage disequilibrium for two SNPs of HLA-DP in 970 individuals. The linkage disequilibrium plot shows r^2^ values between rs3077 and rs9277535. There was not strong LD between rs3077 and rs9277535. D’ = 0.410, r^2^ = 0.130.

Haploview was applied to perform linkage disequilibrium evaluation. As shown in Fig. 1, rs3077 and rs9277535 in HLA-DP were in slight linkage disequilibrium.

### Association analysis of *HLA-DP* polymorphisms with SLE susceptibility

There was a significant difference between the SLE group and the healthy controls in the genotypic distributions of rs3077 and rs9277535. Genotyping and allele modeling showed considerable difference in rs3077. We observed that the rs3077 GA-genotype and the rs9277535 non-GG genotype associated with lower SLE susceptibility (dominant model, rs3077, OR = 0.65, 95%CI = 0.50–0.84, *P* = 0.001; rs9277535, OR = 0.58, 95%CI = 0.44–0.76, *P* < 0.001, respectively). This was supported by the further allele model in which rs3077 allele A and rs9277535 allele A was also showed associated with lower SLE susceptibility (rs3077, OR = 0.74, 95%CI = 0.60–0.91, *P* = 0.004, statistical power = 0.8029; rs9277535, OR = 0.72, 95%CI = 0.59–0.88, *P* = 0.001, statistical power = 0.8970, respectively) (Table 2).

### Association analysis of *HLA-DP* polymorphisms with serum cytokine levels

Analysis of the influence of the two *HLA-DP* polymorphisms on serum cytokine characteristics in SLE patients is illustrated in Table 3. Rs3077 was associated with IL-17, IL-1β, and INF-γ (IL-17: *P* = 0.037, recessive model, *P* = 0.011; IL-1β recessive model, *P* = 0.049; INF-γ: *P* = 0.020, recessive model, *P* = 0.008, respectively) and rs3077 AA genotype had lower IL-17, IL-1β and INF-γ concentrations than the other two genotypes. Meanwhile, serum C3 and C4 concentrations were also affected by rs3077 and rs9277535 polymorphisms (rs3077: C3, *P* = 0.003; rs9277535: C4, recessive model, *P* = 0.019, respectively). Nevertheless, there was no significant association between rs9277535 and IL-17, IL-1β, INF-γ and C3 (IL-17, *P* = 0.949; IL-1β, *P* = 0.569; INF-γ, *P* = 0.895; C3, *P* = 0.299, respectively). And neither rs3077 nor rs9277535 showed association with IL-4, IL-6, IL-10, or IL-23.

### Association analysis of *HLA-DP* polymorphisms with clinical features

Comparing the distribution of genotypic frequencies of *HLA-DP* polymorphisms between positive and negative patients in seven specific clinical symptoms, a significant difference could be observed between rs3077 and cutaneous vasculitis (CV) (χ^2 ^= 10.132, *P* = 0.006) (Table 4). Moreover, as shown in Table 5, a decreased frequency of the minor allele A in CV patients (10.94%) compared with non-CV patients (28.22%) was also observed (χ^2^ = 8.860, *P* = 0.003), which suggests the relationship between the minor A allele of rs3077 and decreased susceptibility to CV in SLE patients (*P* = 0.003, OR = 0.312, 95%CI = 0.140–0.699). In addition, under the dominant model, the minor allele A carriers (GA + AA) exhibit lower risk for CV than those with the GG genotype (GA + AA versus GG: *P* = 0.001, OR = 0.245, 95%CI = 0.098–0.612).

## Discussion

Systemic lupus erythematosus (SLE) is a chronic complex autoimmune disease characterized by autoantibody deposits and involvement of multiple systems^23^. The high heritability, high sibling relapse risk ratio and higher concordance rate in monozygotic twins confirm the genetic effect on the etiology of SLE^24^,^25^. Therefore, the exact genetic pathogenesis of SLE has long been one of the most difficult and intriguing issues.

In the present study, we found two SNPs, rs3077 and rs9277535 of *HLA-DP*, did associate with the SLE susceptibility and the A allele of the two SNPs are potential protective allele in SLE in the Chinese Han population. To our knowledge, *HLA-DP* rs3077 and rs9277535 were not reported to be related to autoimmune diseases (e.g., SLE, RA). It was our study that first investigated the association between the variants of *HLA-DP* and SLE susceptibility in Chinese Han population residing in Southwest China. As previous studies reported, there were some other SNPs of *HLA-DPB1* significantly associated with autoimmune diseases such as rheumatoid arthritis, Wegener’s granulomatosis, systemic sclerosis and infectious diseases like chronic hepatitis B infection^18^,^19^,^20^,^21^,^26^,^27^. Our study suggested that HLA-DP region may contain some genes that have potential protective effects on SLE, which implied *HLA-DP* rs3077 and rs9277535 were likely to represent a shared autoimmune or infectious immune locus.

In addition, a significant positive association was observed between rs3077 with INF-γ, IL-1β and IL-17, and the rs3077 AA genotype had lower INF-γ, IL-1β and IL-17 concentration compared with other two genotypes. Moreover, rs3077 had an association with cutaneous vasculitis (χ^2 ^= 10.132, *P* = 0.006), with the minor allele A carriers showing lower risk of cutaneous vasculitis (*P* = 0.003, OR = 0.312, 95%CI = 0.140–0.699; dominant model: *P* = 0.001, OR = 0.245, 95%CI = 0.098–0.612). Considering that the abnormal expression of Th1, Th2 and Th17 cytokines are important in the pathogenesis of SLE^28^,^29^, it is most likely that the SNP rs3077 was not only associated with SLE susceptibility but also correlated with the levels of some inflammatory cytokines.

*HLA-DP* is mainly expressed on the surface of antigen-presenting cells like macrophages, dendritic cells and B cells, and belongs to HLA α molecules, which can bind and present antigen epitopes to CD4^+^ T helper cells^30^,^31^,^32^. Rs3077 and rs9277535 are located in the 3′ untranslated regions of HLA class II genes *HLA-DPA1* and *HLA-DPB1*, correspondingly^33^,^34^. Therefore, rs3077 and rs9277535 are most likely to affect the disease through influencing the binging and presenting antigen epitopes to CD4^+^ T helper cell. Though without exact evidence, this can be partly supported by the research of O’Brien and his colleagues. In O’Brien’s study, genotype and gene expression data collected and integrated from the normal liver samples obtained from 651 Europeans. They found that rs3077 allele G and rs9277535 allele G were correlated with the down-regulated level of *HLA-DP* mRNA, and allele A was associated with the increased level of *HLA-DP* mRNA in normal human liver tissue^35^. Therefore, considering that allele G of *HLA-DP* rs3077 and rs9277535 have been confirmed predisposed to chronic hepatitis B, carrying allele G of these two SNPs could down-regulate the expression of *HLA-DP*, which turns out to influence the antigen presentation. Compared to HBV infection, SLE is another kind of disease. However, in our present study, allele G was also found predisposed to SLE. These two consistent results suggest that though in different diseases, the role of variants of *HLA-DP* might be the same. Apart from that we have revealed the significant association between the two SNPs of *HLA-DP* and SLE, in the present study, we also found the association between rs3077 and the lower concentration of inflammatory cytokines, as well as the lower risk of cutaneous vasculitis, which might explain partly the function of rs3077 in SLE. Obviously, studies on the structure-function of the two SNPs are still needed. Therefore, the details of the mechanism, including how exactly the gene variants influence the gene expression like HLA-DP mRNA regulation, and the gene function like antigen presentation to CD4^+^ T cells, and so on, would be explored in our future studies. To our knowledge, because these two variants were first reported in SLE, the studies on linkage disequilibrium of HLA-DP in SLE or in autoimmune diseases are inadequate. However, we could take a sight into this issue from the reported linkage disequilibrium of the two variants in other diseases. The allele variant rs3077 in HLA-DPA1 and rs9277535 in HLA-DPB1 were thought that could be independently considered to be possible causative SNPs for HBV in a study which the linkage disequilibrium of multiple-single nucleotide polymorphism genotype data was researched by the textile plot^36^. But in order to clarify the role of these two variants in the numerous alleles, complex structure and the tight linkage disequilibrium of the HLA region, the relationship between these two SNPs and other reported SNPs in HLA would also be discussed in further studies.

In summary, our study firstly found that the two variants (rs3077 and rs9277535) of HLA-DP were associated with decreased SLE susceptibility, and the SNP rs3077 was correlated with the lower IL17, IL-1β and INF-γ concentrations in peripheral blood and reduced susceptibility to CV in SLE patients. This study could provide further evidence to improve our understanding of the exact function of *HLA-DP* in the pathogenesis of autoimmune diseases. It is worthwhile to mention that there are several limitations in our present study. The sample of patients in our study is relatively small and only Han Chinese individuals in western China were included. Therefore, further studies using large sample sizes and other ethnic populations are needed to confirm the results observed in this study.

## Materials and Methods

### Patients and protocol

There were 970 Chinese Han subjects recruited in the present study, including 335 SLE patients and 635 healthy controls from September 2013 to September 2014 in West China Hospital. The SLE patients enrolled met the inclusion criteria as follow: 1) diagnosed as SLE in compliance with the American College of Rheumatology classification criteria for SLE revised in 1997. The patients were all in the active stage of SLE with SLE disease activity index (SLEDAI) >4. 2) hospitalized patients without drug-induced SLE. The main clinical manifestations such as cutaneous vasculitis, arthritis, albuminuria, rash, pleuritis, pericarditis and neurological disorder were obtained retrospectively by reviewing hospital records. The healthy control should meet these inclusion criteria: without any chronic, endemic infectious or autoimmune diseases and with normal physical examination and blood tests.

All the patients have signed the informed consent to participate in this study and consented to sample collection. This study conducted in accordance with the 1975 Declaration of Helsinki and was approved by the Ethics Committee of West China Hospital.

### Serological testing

Laboratory assays of SLE were analyzed by the following methods: ANA, anti-dsDNA and anti-Sm were detected with ANA, anti-dsDNA and ENA reagent kits (Euroimmun company, Germany). Complement 3(C3) and Complement 4(C4) were detected by rate nephelometry on Beckman Coulter IMMAGE 800 immunoassay (Beckman Coulter, Inc, CA, USA). All the tests were conducted according to manufacturers’ instruction.

### *HLA-DP* polymorphism genotyping

Genomic DNA was isolated from the peripheral blood by using Genomic DNA kit (Biotake Corporation, Beijing, China), with the concentration of DNA tested on Nanodrop 2000c spectrophotometer (Thermo Scientific, DE). The polymerase chain reaction-high resolution melting (PCR-HRM) was applied to genotype rs3077 and rs9277535 and the analysis was performed on the Light Cycler 480 (Roche Diagnostics, Penzberg, Bavaria, Germany). SNP genotyping was performed in a 20 μL reaction system which contains 10 μL Roche Master Mix (Roche Applied Science) (comprising FastStart Taq DNA Polymerase and the High Resolution Melting Dye in a reaction buffer), 2.4 μL 25 mM MgCl_2_, 0.2 μL 10 mmol/L Forward Primer, 0.2 μL 10 mmol/L Reverse Primer, 6.2 μL deionized water and 1 μL DNA sample as recommended by the manufacturer. The whole genotyping process was performed under the following conditions: 95 °C for 10 min in the initial denaturation step followed by 50 cycles of 95 °C for 15 s, touchdown cycling (decreasing 1 °C/cycle), 65–55 °C for 10 s, and 72 °C for 10 s in the annealing step and then denatured at 95 °C for 1 min and cooled to 40 °C for 1 min after the amplification phase. After HRM analyses, melting process and cooling, the results were analyzed by the corresponding Gene Scanning Software v1.2 (Roche Diagnostic).

### Cytokine measurement

Randomly 93 samples of SLE patients were chosen to test the serum cytokine. The serum concentrations of cytokines including IL-1β, IL-4, IL-6, IL-10, IL-17, IL-23, TNF-α and INF-γ were estimated. They were quantitatively determined by Bio-Plex Pro™ Human Inflammation Assays. Procedure was performed according to the manufacturer’s instructions. Blood sampling for assessing serum levels of cytokines and other laboratory investigations were performed at the same time of clinical examination and assessment of SLE disease activity.

### Statistical analysis

Statistical power was calculated by a software “PS: Power and Sample size Calculation” (http://biostat.mc.vanderbilt.edu/wiki/Main/PowerSampleSize). The Hardy-Weinberg equilibrium (HWE) was evaluated for each polymorphism independently. Mean ± SD, median and interquartile was used to describe the continuous variables with normal and skewed distribution, respectively. Student’s t test or Mann-Whitney U test were applied to compare demographic and clinical data between groups as appropriate. Allele case-control comparisons were analyzed by pearson’s chi-square test or Fisher’s exact test. Association of SNPs with susceptibility of SLE was assessed by figuring out the odds ratio (OR) and 95% Confidence Interval (CI). The following analytic methods were used when it came to compare the subjects from two groups: allelic frequency distribution of the two groups (allele A versus allele B, A as the major allele, B as the minor allele, this applied to the following methods); dominant model (AB + BB versus AA); recessive model (AA + AB versus BB). All statistical analyses were executed applying the Statistical Package for the Social Sciences (SPSS, SPSS Inc., Chicago, IL, USA), version 19.0. A two-sided *P* value < 0.05 was considered to be statistically significant.

## Additional Information

**How to cite this article:** Zhang, J. *et al*. Genetic Polymorphisms of rs3077 and rs9277535 in HLA-DP associated with Systemic lupus erythematosus in a Chinese population. *Sci. Rep.* **7**, 39757; doi: 10.1038/srep39757 (2017).

**Publisher's note:** Springer Nature remains neutral with regard to jurisdictional claims in published maps and institutional affiliations.

## Figures and Tables

**Table 1:** The demographic and clinical characteristics of the subjects recruited in the study.

Characteristics	SLE	Control	*P* value
Number of subjects	335	635	—
Age, mean ± SD (years)	35.77 ± 13.25	36.28 ± 13.43	0.795
Male (%)/Female (%)	37 (11.04)/298 (88.96)	80 (12.60)/555(87.40)	0.480
Disease duration, mean ± quartile interval (months)	36.00 (12.00–96.00)	—	—
SLEDAI scores, mean ± SD	11.76 ± 5.53	—	—
ANA-positive(%)	308 (91.94)	—	—
anti-dsDNA-positive (%)	120 (35.82)	—	—
anti-Sm-positive(%)	96 (28.66)	—	—
C3, mean ± SD(g/L)	0.67 ± 0.30	—	—
C4, mean ± SD(g/L)	0.15 ± 0.08	—	—
Cutaneous vasculitis (%)	32 (9.55)	—	—
Arthritis (%)	132 (39.40)	—	—
Albuminuria (%)	250 (74.63)	—	—
Rash (%)	100 (29.85)	—	—
Pleuritis (%)	5 (1.49)	—	—
Pericarditis (%)	5 (1.49)	—	—
Neurological disorder (%)	33 (9.85)	—	—

Abbreviations: SLE, systemic lupus erythematosus; SD, standard deviation; SLEDAI, SLE disease activity index; ANA, antinuclear antibody; anti-dsDNA, double-stranded DNA antibody; anti-Sm, Smith antibody; C3, complement 3; C4, complement 4.

**Table 2:** Genotype distributions of *HLA-DP* in SLE patients and controls in Chinese Han population.

SNPs	Model	Genotype	SLE (n = 335)		Controls (n = 635)		OR(95%CI)	*P* value
			N	%	N	%		
rs3077		GG	182	54.33	276	43.46	1	—
		GA	128	38.21	301	47.40	0.65(0.49–0.85)	**0.002**
		AA	25	7.46	58	9.13	0.65(0.39–1.08)	0.094
	Dominant	GA + AA/GG	—	—	—	—	0.65(0.50–0.84)	**0.001**
	Allele	G	492	73.43	853	67.17	1	—
		A	178	26.57	417	32.83	0.74(0.60–0.91)	**0.004**
rs9277535		GG	166	49.55	231	36.38	1	—
		GA	127	37.91	314	49.45	0.56(0.42–0.75)	**<0.001**
		AA	42	12.54	90	14.17	0.64(0.42–0.98)	**0.040**
	Dominant	GA + AA/GG	—	—	—	—	0.58(0.44–0.76)	**<0.001**
	Allele	G	459	68.51	776	61.10	1	—
		A	211	31.49	494	38.90	0.72(0.59–0.88)	**0.001**

Abbreviations: SLE, systemic lupus erythematosus; OR, odds ratio; CI, confidence interval.

**Table 3:** Association between HLA-DP polymorphisms with serum cytokine characteristics in SLE patients.

SNPs	Genotype	C3	*P* Value	C4	*P* Value	IFN-γ	*P* Value	IL-1β	*P* Value	IL-17	*P* Value	IL-6	*P* Value	IL-10	*P* Value	TNF-α	*P* Value
		Mean ± SD(g/L)		Mean ± SD(g/L)		Mean ± SD(pg/ml)		Mean ± SD(pg/ml)		Mean ± quartile interval (pg/ml)		Mean ± quartile interval (pg/ml)		Mean ± quartile interval (pg/ml)		Mean ± quartile interval (pg/ml)	
rs3077	GG	0.63 ± 0.27		0.14 ± 0.07		512.49 ± 199.16		4.07 ± 3.08		117.07(79.65–134.19)		28.53(21.23–40.80)		5.67(4.39–7.86)		4.45(1.60–9.72)	
	GA	0.74 ± 0.33	**0.003**	0.16 ± 0.09	0.118	551.27 ± 193.35	**0.020**	4.75 ± 3.06	0.164	106.05(78.06–167.36)	**0.037**	29.71(21.42–44.58)	0.685	5.67(4.39–6.98)	0.887	4.83(1.65–9.04)	0.923
	AA	0.58 ± 0.29		0.13 ± 0.06		359.27 ± 203.34		2.82 ± 2.50		63.67(34.30–100.84)		29.71(9.80–35.75)		6.08(1.34–10.75)		3.38(2.88–29.28)	
	GG	0.63 ± 0.27		0.14 ± 0.07		512.49 ± 199.16		4.07 ± 3.08		117.07(79.65–134.19)		28.53(21.23–40.80)		5.67(4.39–7.86)		4.45(1.60–9.72)	
	GA + AA	0.71 ± 0.33	0.051	0.15 ± 0.09	0.830	509.03 ± 209.49	0.935	4.32 ± 3.03	0.693	100.83(64.75–157.17)	0.488	29.71(19.29–42.00)	0.871	5.77(4.39–7.22)	0.674	4.38(2.16–9.19)	0.929
	AA	0.58 ± 0.29		0.13 ± 0.06		359.27 ± 203.34		2.82 ± 2.50		63.67(34.30–100.84)		29.71(9.80–35.75)		6.08(1.34–10.75)		3.38(2.88–29.28)	
	GA + GG	0.68 ± 0.30	0.748	0.15 ± 0.08	0.448	530.93 ± 196.18	**0.008**	4.39 ± 3.07	**0.049**	114.33(77.74–157.17)	**0.011**	28.83(21.18–43.60)	0.454	5.67(4.39–7.53)	0.924	4.76(1.6–9.72)	0.591
rs9277535	GG	0.66 ± 0.29		0.14 ± 0.07		519.99 ± 190.57		3.96 ± 2.58		110.24(76.79–127.48)		26.19(20.55–39.60)		6.38(4.61–7.47)		4.4(1.44–9.74)	
	GA	0.66 ± 0.27	0.299	0.14 ± 0.07	0.060	508.42 ± 226.99	0.895	4.58 ± 3.58	0.569	106.05(63.67–165.86)	0.949	48.34(34.36–103.58)	0.806	5.93(5.57–7.66)	0.633	4.76(3.11–21.07)	0.824
	AA	0.73 ± 0.36		0.18 ± 0.12		489.50 ± 174.72		3.78 ± 2.50		104.17(77.42–132.41)		32.08(18.42–42.79)		5.87(4.39–9.00)		4.16(2.05–7.38)	
	GG	0.66 ± 0.29		0.14 ± 0.07		519.99 ± 190.57		3.96 ± 2.58		110.24(76.79–127.48)		26.19(20.55–39.60)		6.38(4.61–7.47)		4.4(1.44–9.74)	
	GA + AA	0.68 ± 0.31	0.698	0.15 ± 0.09	0.805	503.86 ± 214.16	0.708	4.39 ± 3.35	0.501	105.45(69.88–162.32)	0.827	30.01(19.29–45.12)	0.369	5.36(4.06–7.65)	0.731	4.83(2.87–9.20)	0.565
	AA	0.73 ± 0.36		0.18 ± 0.12		489.50 ± 174.72		3.78 ± 2.50		104.17(77.42–132.41)		32.08(18.42–42.79)		5.87(4.39–9.00)		4.16(2.05–7.38)	
	GA + GG	0.66 ± 0.29	0.151	0.14 ± 0.07	**0.019**	514.06 ± 208.78	0.689	4.28 ± 3.13	0.587	107.81(67.85–142.21)	0.881	28.83(19.72–40.38)	0.599	5.67(4.28–7.62)	0.735	4.45(1.99–9.74)	0.925

**Table 4:** Association between *HLA-DP* polymorphisms with clinical characteristics in SLE patients.

Clinical feature	Rs3077					Rs9277535				
	Genotype frequency(n(%))			χ^2^	p-Value	Genotype frequency(n(%))			χ^2^	p-Value
	GG	GA	AA			GG	GA	AA		
Cutaneous vasculitis	26(81.25)	5(15.63)	1(3.13)	10.132	0.006	19(59.38)	9(28.13)	4(12.50)	1.575	0.460
Arthritis	78(59.09)	42(31.82)	12(9.09)	4.012	0.134	68(51.52)	48(36.36)	16(12.12)	0.337	0.846
Albuminuria	141(56.40)	89(35.60)	20(8.00)	2.915	0.241	126(50.40)	97(38.80)	27(10.80)	2.721	0.263
Rash	58(58.00)	34(34.00)	8(8.00)	1.070	0.614	50(50.00)	39(39.00)	11(11.00)	0.319	0.865
Pleuritis	2(40.00)	1(20.00)	2(40.00)	5.279	0.062^a^	2(40.00)	2(40.00)	1(20.00)	0.891	0.824^a^
Pericarditis	2(40.00)	1(20.00)	2(40.00)	5.279	0.062^a^	2(40.00)	2(40.00)	1(20.00)	0.891	0.824^a^
Neurological disorder	16(48.48)	13(39.39)	4(12.12)	1.565	0.423^a^	11(33.33)	16(48.48)	6(18.18)	4.225	0.113^a^

^a^Fisher’s exact test.

**Table 5:** Distribution of the genotype and allele frequencies of rs3077 polymorphism in cutaneous vasculitis (CV) and non-CV patients.

		CV(n = 32),n(%)	Non-CV(303),n(%)	χ2	p-Value	OR (95%CI)
Genotype	GG	26(81.25)	156(51.49)	10.132	**0.006**	NA
	GA	5(15.63)	123(40.59)			
	AA	1(3.13)	24(7.92)			
Allele	G	57(89.06)	435(71.78)	8.860	**0.003**	—
	A	7(10.94)	171(28.22)			0.312(0.140–0.699)
Dominant model	GG	26(81.25)	156(51.49)	10.334	**0.001**	—
	GA + AA	6(18.75)	147(48.51)			0.245(0.098–0.612)
Recessive model	AA	1(3.13)	24(7.92)	0.964	0.490	—
	GG + GA	31(96.88)	279(92.08)			2.667(0.349–20.396)

Abbreviations: CV: cutaneous vasculitis; OR, odds ratio; CI, confidence interval; NA: not available.
